# GASOLINE: a Greedy And Stochastic algorithm for Optimal Local multiple alignment of Interaction NEtworks

**DOI:** 10.1371/journal.pone.0098750

**Published:** 2014-06-09

**Authors:** Giovanni Micale, Alfredo Pulvirenti, Rosalba Giugno, Alfredo Ferro

**Affiliations:** 1 Department of Computer Science, University of Pisa, Pisa, Italy; 2 Department of Clinical and Molecular Biomedicine, University of Catania, Catania, Italy; University of East Piedmont, Italy

## Abstract

The analysis of structure and dynamics of biological networks plays a central role in understanding the intrinsic complexity of biological systems. Biological networks have been considered a suitable formalism to extend evolutionary and comparative biology. In this paper we present GASOLINE, an algorithm for multiple local network alignment based on statistical iterative sampling in connection to a greedy strategy. GASOLINE overcomes the limits of current approaches by producing biologically significant alignments within a feasible running time, even for very large input instances. The method has been extensively tested on a database of real and synthetic biological networks. A comprehensive comparison with state-of-the art algorithms clearly shows that GASOLINE yields the best results in terms of both reliability of alignments and running time on real biological networks and results comparable in terms of quality of alignments on synthetic networks. GASOLINE has been developed in Java, and is available, along with all the computed alignments, at the following URL: http://ferrolab.dmi.unict.it/gasoline/gasoline.html.

## Introduction

The structure and the dynamic of biological networks arise from interactions among molecules within the cell. Biological functions are obtained by the collaborative action of a number of cellular constituents, such as proteins, DNA, RNA and other small molecules [1]. Consequently, reductionism focusing only on the study of individual molecules and their limited interactions has shown its inadequacy in providing a comprehensive picture of living cells. Recently, high-throughput techniques for measuring protein-protein interactions (PPIs) have been introduced. Two-hybrid screening [2] and coimmuno-precipitation followed by mass-spectrometry [3] allowed the systematic study of protein interactions on a global scale. Extensive mining of scientific literature produces a variety of known biological interactions [4], [5]. Several public and commercial databases, such as BioGRID [6], DIP [7], STRING [8], MINT [9], Yeast Proteome Database (YPD) [10] and Pathway Commons [11] collect specific knowledge in this area. The rapidly growing number and size of biological networks raises an important question on how can we make use of this network data to infer novel biological insights. A retrospective view of the recent history of molecular biology research shows that most of the attention has been devoted to sequence analysis. This indeed represents a fundamental level of biological investigation and for a long time has been the basis of evolutionary studies [12]. Recently, it has been shown that a system oriented approach to the study of biological phenomena may be more appropriate. In analogy with multiple sequence alignment, in which relevant functional parts of a single sequence are highlighted, common patterns in biological networks of different species provide an effective means of identifying functional modules (e.g., signaling pathways or protein complexes) conserved through evolution. Several research groups have proposed techniques to systematically analyze and compare biological networks. A typical network analysis includes (i) network querying [13]–[17], which is commonly used to find structural (possibly) approximated motifs and to establish whether such motifs are functional (over-represented); (ii) global and local network alignment [18]–[23], to understand if some functional complexes are conserved through species to infer evolutionary relationships among networks. Local network alignment approaches based on Hidden Markov Models have been proposed [24], [25]. However, their action has been restricted to the identification of shared paths.

In this paper, we present GASOLINE (Greedy And Stochastic algorithm for Optimal Local multiple alignment of Interaction NEtworks), a novel algorithm for protein networks alignment based on iterative sampling [26] in connection with a greedy strategy. GASOLINE is inspired by the work of [27] and implements a seed-and-extend approach to extract shared complexes among a set of protein-interaction networks. The algorithm starts by identifying a set of similar nodes, one from each network, by using a Gibbs Sampling strategy. Then, a similar technique is applied to extend the alignment. This step is iterated until the local density of the aligned subgraphs, measured through a properly defined *degree ratio*, increases. The algorithm iterates the above steps producing a set of local networks alignments (where each local alignment consists of a set of similar, in terms of both sequence and structure similarity, subgraphs). At the end of the process, each set of local networks alignments is ranked according to an index called Index of Structural Conservation. We extensively tested GASOLINE on: (i) a set of 25 biological networks drawn from STRING [8]; (ii) a set of artificially generated networks using the NAPABench tool [28].

We compared our system with a selected list of state-of-the-art methods such as SMETANA [23], IsoRank-N [22] and NetworkBlast-M [19]. The experimental results show that GASOLINE outperforms the compared systems yielding the best results in terms of both quality (i.e. precision and recall) and running time. The method is very general and can be applied to any type of networks (e.g. social, web, molecular) by using appropriate label and graph similarities.

## Methods

Given 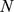 weighted biological networks, where weights are probabilities expressing the reliability of pairwise protein relations (i.e. protein interactions in the case of PPI), informally, the local alignment of biological networks aims at finding a set of 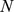 regions (in our modeling subgraphs having the same number of proteins), one from each network, that are conserved in their sequence and interaction pattern.

Such a problem is related to subgraph isomorphism, which is known to be NP-complete, therefore heuristics are needed. GASOLINE is able to produce an approximate solution through a stochastic-greedy strategy consisting of two phases.

During the first step called *bootstrap phase*, we look for orthologous proteins across the networks and build a set of seeds. The set of seeds initially consists of 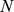 proteins, one from each network, and includes all the starting nodes of the suboptimal local network alignment we are searching for.

The second step, called *iterative phase*, repeatedly either adds (extension step) or removes (removal step) nodes in the network alignment, trying to maximize the final alignment score. Each extension step adds, in each network, a single node to the corresponding seed. During the extension step the seeds grow up producing a set of 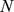 subgraphs, one from each network. The extension process is regulated by a properly defined degree ratio measuring the average density of the aligned subgraphs with respect to their neighbors in the networks. The extension is performed until this degree ratio increases.

Each removal step replaces from the current alignment the set of proteins (one from each network) providing the minimum score.

The initial phase and each extension step are performed through an iterative sampling. Consequently, different iterations of the algorithm may produce different local alignments. GASOLINE iterates the above steps producing a set of local networks alignments. Those local alignments are ranked according to an Index of Structural Conservation (ISC) score combining topology and sequence similarity.

GASOLINE implements preprocessing and post-processing steps. During preprocessing, the search space for potential seeds is reduced. This is obtained by marking only proteins having orthologs in all aligning networks and with a significant interaction degree in each network.

**Figure 1:**
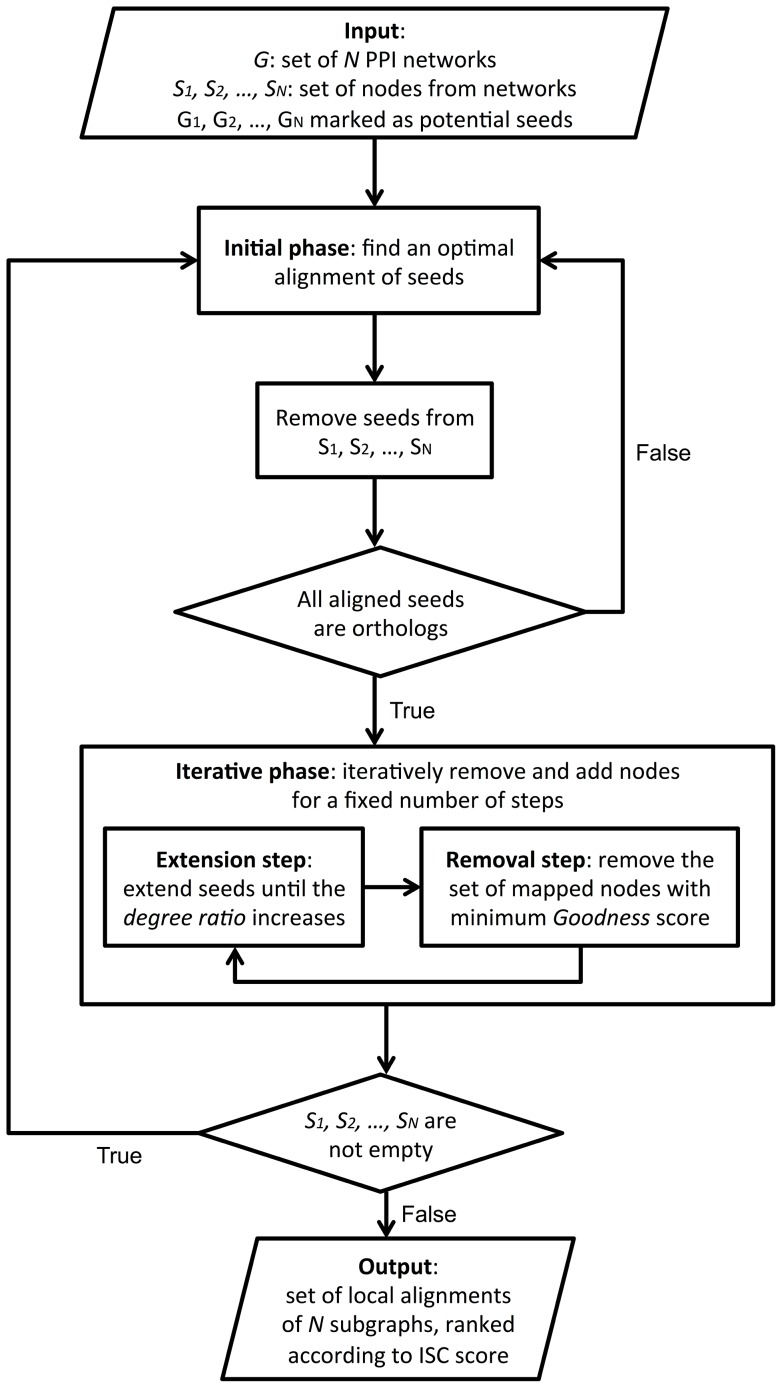
General description of GASOLINE.

All marked nodes in each network 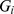 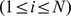 are added to a set called 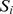. These sets will be used in the initial phase and will be updated at each iteration. Finally, during post-processing, the final set of local alignments returned by GASOLINE is filtered by removing highly overlapping complexes. Flowchart in Figure 1 provides a general description of GASOLINE.

The computational complexity analysis assumes that all networks have the same number 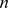 of nodes and each iteration of the algorithm returns an alignment of subgraphs having size 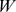. The complexity is given by 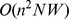. Assuming that 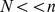 the complexity can be rewritten as 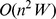. The worst case applies when networks are dense and very similar implying that the average size 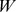 of aligned complexes is 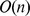. In this case the algorithm has an execution time 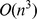. In the average case we can assume that the size of aligned complexes is still function of 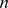 in particular 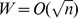. Therefore the complexity will be 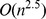. The best case applies when 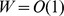. In this case the complexity will be 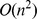 (please refer to File S1 for the details on the complexity analysis). Next section contains a more detailed description of the algorithm.

### The algorithm

Let 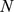 be the number of aligning networks. Each iteration of GASOLINE starts by searching an alignment of nodes (one node from each network) which can be viewed as the seeds of a candidate local alignment. Candidate proteins for the initial alignment are drawn from the sets of marked nodes 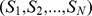, built in the preprocessing step. Once this step has produced a set of orthologous proteins, the iterative phase begins. Through this phase the seeds set is extended producing a list of subgrahps one from each subgraphs.

At the end of the iterative phase the aligned seeds are removed from the sets 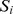 (in order to guarantee termination) and the process starts again from the bootstrap phase with new seeds proteins chosen in 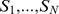.

#### Bootstrap phase

The search for an initial set of seeds is performed by a Monte Carlo Markov Chain in connection with a Gibbs Sampling algorithm. The Gibbs sampling builds a chain, where each state represents a combination (i.e. alignment) of 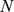 proteins, one from each network. First, a random initial state is selected. Then, the sampling method iteratively performs a transition from a state to another, by replacing a randomly chosen protein of the current alignment with a protein of the same network, according to a properly defined transition probability distribution. By iterating this sampling procedure a sufficient number of times, we eventually achieve a good alignment of seeds.

The transition probability is defined on top of a *Similarity Score*. Given two proteins 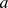 and 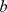, we define their similarity score 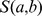 as either their Bit Score or the inverse of their BLAST E-value [29]. Let 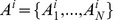 be the alignment of proteins at the 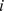-th iteration of Gibbs sampling and suppose we remove the node 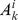 from it. Let 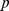 be a candidate protein replacing 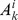. The similarity score of 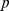 is defined as the product of all similarity scores between 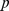 and the proteins still belonging to the alignment: 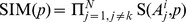. The transition probability in 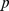 is then computed by using such the similarity scores as follows:
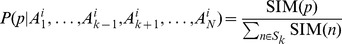



Finally, the alignment score is defined as the sum-of-pairs of similarity scores between the aligned proteins:
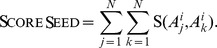



At the end of the bootstrap phase the alignment of seeds maximizing the sum-of-pairs score over all the iterations of the Gibbs sampling is chosen.

#### Extension of current seeds

Let 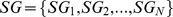 be an alignment of 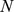 subgraphs, one for each network and 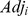 the set of nodes adjacent to one or more nodes in 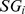. The goal of each extension step is to find an alignment 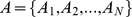 of 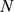 proteins where 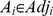, and extend each 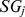 with 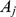 and the edges connecting 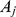 with the remaining nodes in 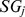.

**Figure 2:**
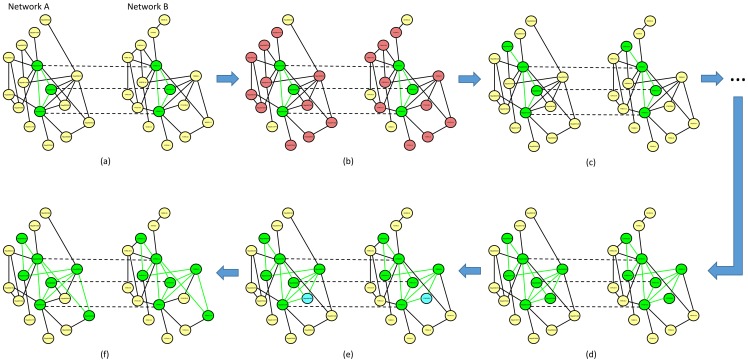
A toy example of extension and removal phases of GASOLINE algorithm in a pairwise alignment instance. (a) The nodes of the two aligned subgraphs are highlighted in green. (b) In red are highlighted those adjacent nodes of current alignment which will be explored by the sampling algorithm during the next step of iterative phase. (c) At the end ot such iteration the alignment will be extended with a new node in each network. (d) Once the iterative phase completes, it gives as a result the aligned subgraphs highlighted in green. In (e) the removal step identifies the cyan nodes as those contributing less to the alignment score. (f) These will be replaced with those capable to increase the alignment score.

Figure 2 shows a demo with two aligning networks. In Figure 2 (a) the current alignment 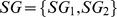, consisting of two subgraphs composed by three nodes, is highlighted in green, with dashed lines connecting aligned proteins. Figure 2 (b) highlights in red all the nodes in 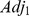 and 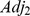. In Figure 2 (c) the new alignment of subgraphs yielded after a single extension step is shown in green.

Each extension step is performed through an iterative sampling similar to the one described above, where a state of the Markov chain represents an alignment of 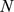 nodes, one for each set 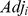.

Again, the initial state of the chain is randomly selected. Then, a series of transitions from a state to another one is made, by replacing a randomly chosen protein of the current alignment with a node of the same network in the corresponding adjacent set. The transition probabilities are computed by considering sequence similarity in connection with neighborhood similarity.

Let 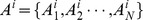 be the alignment of proteins at the 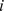-th iteration of Gibbs sampling. Suppose we remove protein 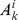 from 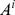 and let 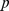 be a protein of the same network candidate to replace it.

The Similarity Score of 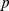 takes into account both the orthology relation and the topological similarity between 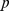 and proteins in 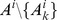.

The orthology score of 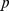 which takes into account the sequence similarity, is defined as in the bootstrap phase: 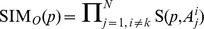.

Concerning the topology similarity, we build a vector 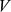, called *topology vector*, storing the weights of the edges linking 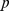 to the nodes of 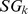. If there is no link between two proteins the weight is set to 0. Likewise, we build a topology vector for all the proteins in 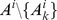.

Given two proteins 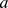 and 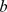, and their topology vectors 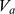 and 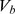, the topology similarity score is the scalar product of the two vectors: 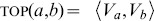. The topology similarity score of 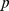 is then defined as: 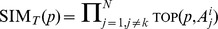. The overall similarity Score of 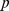 can the be computed as: 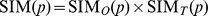. By normalizing in the range 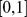 we obtain the transition probability of 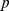:
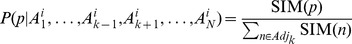



The alignment score is calculated in terms of orthology and topology similarity by the sum-of-pair of the pairwise alignments:
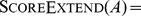


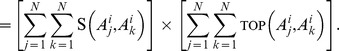



At the end of Gibbs sampling, the alignment with highest sum-of-pair score is selected for the extension of subgraphs in 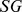.

The extension of subgraphs mainly depends on a degree ratio of the alignment which evaluates the local density and the modularity of aligned subgraphs with respect to their neighborhood. Given an aligned subgraph 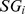, the degree ratio of 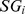 is the number of edges linking nodes within 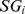 over the sum of the degrees of nodes in 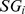. Then, the degree ratio of a subgraph alignment 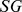 is the average degree ratio of aligned subgraphs in 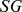. The extension process is repeated until the following two properties hold: (i) all mapped proteins are in orthology relation (w.r.t. BLAST E-values or Bit Scores); (ii) the degree ratio of 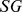 strictly increases.

#### Removal step

In the removal step, we discard from the current alignment a set of mapped proteins which give a minimal contribution to alignment quality. Such a step tries to refine the topology of the aligned subgraphs and therefore does not take into account the sequence similarity. The reason behind this choice is that during the extension steps the subgraph topology conservation intrinsically decreases since no backtracking is performed. Such a step deals with such an issue by making use of a measure called Goodness score.

Let 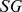 be the current subgraph alignment and let 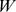 be the number of proteins in each aligned subgraph. We can represent 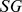 as a 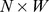 matrix, where each column contains mapped proteins across all the networks. The goal of this step is to delete the column minimizing Goodness. We define the Goodness of a generic protein 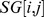 of alignment 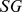 as the ratio between the internal degree of 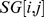, i.e. the number of links connecting 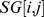 to the remaining nodes in the aligned subgraph, and its node degree. The Goodness of column 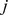 is the product of the Goodness scores of all its proteins:
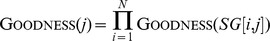



Each removal step deletes from the current alignment the nodes corresponding to the column with the minimum Goodness score. However, such proteins could be added again to the alignment, in some future extension steps. In Figure 2 (d)–(e) we report a toy example of the removal step. Figure 2 (d) consists of the current local alingment identified through the iterative step; In (e) the removal step identifies two cyan nodes as those giving a minimal contribution to the alignment score; In (f) the nodes are replaced with to different nodes increasing the alignment score.

Notice that, the topology similarity score between proteins in the Gibbs sampling algorithm of the extension process is defined in order to reward structural conservation, edge weights and density of the aligning subgraphs. So, as long as the extension process continues, the degree ratio increases. However, it tends to reach local maxima, so the goal of the refinement phase is to try to shift from these local maxima, in order to reach a better approximation of the global maximum.

#### Final alignments ranking

Once the algorithm completes the extraction of conserved subgraphs, GASOLINE ranks all the alignments through a score called *Index of Structural Conservation* (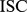) which measures its quality in terms of topology and sequence similarity. Let 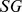 be the current subgraph alignment and let 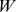 be the number of nodes in each aligned subnetwork. 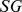 can be represented as a matrix with 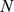 rows and 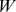 columns, where the 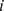-th row stores proteins of the aligned subgraph 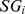. The structural similarity score between two aligned subgraphs, 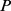 and 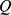 (i.e. two rows of the above matrix), measures the similarity between the topology vectors of the corresponding proteins in the current mapping. Let 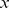 and 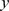 be two nodes and 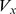 and 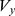 their topology vectors. 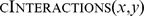 denotes the percentage of entries in 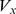 and 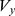 that are either both null or both different from zero (consisting of conserved links in both species):
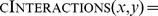


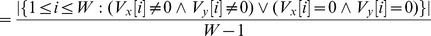



The pairwise structural similarity score 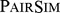 between 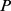 and 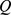 is given by:
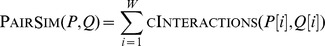
where 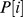 and 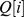 are the matched nodes in 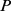 and 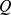 respectively. The structural similarity score of alignment 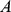, 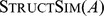, can be defined as the sum-of-pair of all pairwise structural similarity scores:



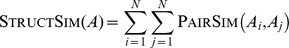



According to this definition the maximum 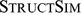 value is 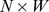, achieved by 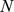 perfectly aligned cliques. Finally, the 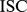 of an alignment A can be defined as the normalization of 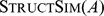 in the 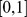 interval:
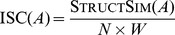



#### Postprocessing

The final set of local alignments returned by GASOLINE is post-processed to filter out highly overlapping complexes. Alignments are sorted according to their size and 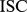 score. Let 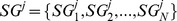 the local alignment of rank 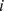 in the sorted list. For each subnetwork 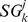 of the alignment 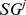, 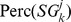 denotes the percentage of proteins in 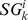 observed in the previous 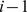 alignments. Let 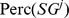 the average value of 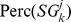 across all the networks. If 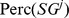 is above a given threshold the alignment is discarded.

## Results and Discussion

We performed a set of experiments on synthetic and real biological networks to asses the performance of GASOLINE. All tests have been performed in a Intel Core i7-2670 2.2Ghz CPU with a RAM of 8 GB.

### Data Description and Experimental Setup

Synthetic Biological networks were generated using NAPAbench [28], a large-scale network alignment benchmark for generating families of evolutionary related synthetic PPI networks, evolved from a common ancestor, according to a given phylogenetic tree. It has been recently used as a framework to compare the accuracy and the scalability of different alignment algorithms [23], [28].

Real Biological networks were taken from STRING (version 9.0) [8], a database of known and predicted PPIs, collected from different high-throughput experiments, coexpression data and publications. For every examined species, we filtered the set of interactions, considering only experimentally supported interactions (i.e. those with positive values on “Experimental” field). We point out that this kind of protein interactions can also result from experimental knowledge transferred from one species to another.

Three different case studies have been examined:

1. Pairwise and multiple alignments of synthetic networks;
2. 6-way alignment of real PPI eukaryotic networks;
3. 25-way alignment of real PPI vertebrata networks.

In case studies a) and b) we compared our method against three different global and local multiple network alignment algorithms: SMETANA [23], IsoRankN [22] and NetworkBLAST-M [19]. To our knowledge, the first two methods are the best global many-to-many aligners of two or more species, while NetworkBlast-M represents the state-of-the-art for the local alignment problem. We chose both global and local alignment methods in order to (i) highlight the ability of GASOLINE to correctly map many proteins of different species as a good global aligner does; (ii) find many conserved complexes as a good local aligner does. In our experiments we ran IsoRankN with 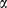 = 0.7 and 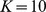 and we used the restricted-order version of NetworkBLAST-M for computational reasons. To compute similarities between proteins, we used Blast bit scores for GASOLINE, SMETANA and IsoRankN, and Blast E-values for NetworkBLAST-M.

We used several measures to evaluate the specificity, the sensitivity and the functional consistency of the alignment algorithms, both for synthetic and for real biological networks, following the methodology described in [28]. We also tested the robustness of the analyzed methods in case of low sequence similarity between homologous proteins and the scalability with respect to the number and the size of aligned networks.

In case study c) we tested the ability of GASOLINE to find highly conserved complexes across many species in reasonable time. Starting from the information about orthologous groups (COG, KOG and NOG) obtained from the STRING database, we computed the Jaccard similarity coefficient [30] between the sets of two proteins' orthology groups. That is defined as the number of common groups divided by the cardinality of the union of the two sets.

The algorithm needs a few parameters to be set out:

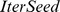
: number of iterations of Gibbs sampling in the bootstrap phase;

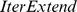
: number of iterations of Gibbs sampling in the extension step;

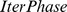
: number of iterations of each iterative phase;

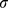
: threshold value for the degree of candidate seed nodes;

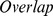
: threshold value for overlap percentage;

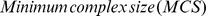
: minimum number of proteins of a conserved complex;

Some of these parameters were established experimentally:

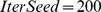
;

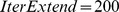
;

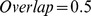
;

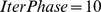
;

Notice that, some parameters are strictly related to the stochastic nature of the algorithm (i.e. IterSeed, IterExtend, IterPhase). Such parameters have been determined in connection to the convergence of the algorithm on the network instances tested. Therefore, we suggest these default parameters since are enough to yield good alignment results.

The threshold parameter (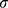) for the seed selection represents a tradeoff between speed and accuracy of our method. In order to maximize the accuracy and the coverage of GASOLINE, its value has been set to 1 in all comparisons. This means that no filtering on the nodes has been applied for the networks alignment. However, we give the possibility to the users to increase the value of such parameter for large input instance, as we did in third case study for the 25-way alignment. Concerning the 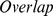 parameter, it allows to filter the output produced by the algorithm. We chose an intermediate value (0.5) for this parameter. However, the user can vary this parameter to tune the number of subgraphs alignments that GASOLINE gives as output.

As regards the 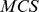 parameter, this essentially allows to set the smallest size of subgraphs alignments. In our experiments, we set this parameter to the minimum value (1), in order to maximize protein coverage, since we are comparing our method with global alignment algorithms too. Unlike the threshold parameter for the seed selection, it does not affect the running time of GASOLINE, since it concerns the postprocessing phase.

### Case study 1: alignment of synthetic networks

We first assessed the performance of the proposed method on different datasets of synthetic similar PPI networks generated with NAPAbench [28]. We considered three different partitions of datasets. Each partition consists of three families of aligning networks, generated using three different network growth models, i.e. *duplication-mutation-complementation* model (DMC) [31], *duplication with random mutations* model (DMR) [32], [33] and *crystal growth* model (CG) [34]. From now on, we will denote them as DMC, DMR and CG families. We set 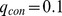 and 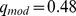 for DMC, 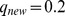 and 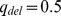 for DMR and 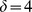 for CG.

The first partition is formed by families of 2 closely related networks, evolved from a common ancestor with 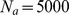 nodes. The families of the second partition consists of 4 evolutionary distant networks, with a common ancestor of 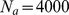 nodes. In the last partition, each family contains 8 networks with different evolutionary distances, generated from a common ancestor of 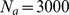 nodes.

**Figure 3:**
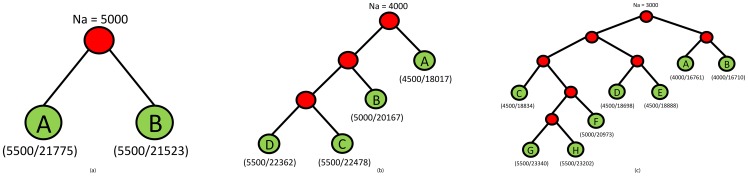
Phylogenetic trees for the synthetic networks generated using NAPAbench. (a) 2-way alignment, (b) 4-way alignment, (c) 8-way alignment. Below each leaf node, the number of nodes and the average number of edges across the CG, DMC and DMR families of the corresponding network are shown in parenthesis.

Figure 3 depicts the phylogenetic trees used for the families of each partition. All the branches of the phylogenetic tree have weight 500, meaning that each node of the tree (except the root) is a network obtained from the parent node by adding 500 nodes according to the growth model used.

To measure the overall accuracy of the proposed methods, we used functional groups associated by NAPAbench to each protein of the aligning networks. We call equivalence class a set of proteins of different species (one or more for each network), which are mapped together by a given algorithm. An equivalence class is claimed as correct if all the included nodes belong to the same functional group. For each method we computed three different quality measures:

- *Specificity* (SPE): the relative number of correct equivalence classes;
- *Correct nodes* (CN): the total number of proteins assigned to correct equivalence classes;
- *Mean normalized entropy* (MNE): the mean normalized entropy of the predicted equivalence classes. Given an equivalence class 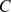, the normalized entropy of 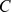 is computed by:

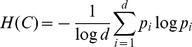
where 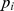 is the fraction of proteins in 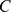 that belong to the 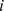-th functional group and 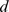 is the number of different functional groups.

CN reflects the sensitivity of the method, while MNE measures the consistency of the predicted alignments. For SMETANA and IsoRankN we considered only equivalence classes that contain at least one node from each species.

Tables 1, 2 and 3 summarize the values of SPE, CN and MNE of the proposed methods for all the alignments of 2, 4 and 8 networks, respectively. Each table reports the results obtained for DMC, DMR and CG families. In all cases SMETANA has the highest sensitivity, recovering a high number of CN. However, our method resulted more precise, especially in the 8-way alignment, resulting in a higher specificity and a lower rate of false positives. The lower sensitivity of GASOLINE is due to the fact that our method is based on 1-to-1 mapping, while SMETANA performs a many-to-many alignment. The other two methods generally exhibit lower specificity, sensitivity and consistency than SMETANA and GASOLINE. Interestingly, the specificity of GASOLINE remains very high (around 90%), even though the number of networks increases, while the accuracy of all the other algorithms tends to decrease. In particular, the accuracy of NetworkBLAST-M falls down from pairwise to 8-way alignment, going from 88% to 4%.

**Table 1:** Performance of alignment algorithms for pairwise alignments of synthetic PPI networks (CG = crystal growth model, DMC = duplication-mutation-complementation model, DMR = duplication with random mutations model).

	CG			DMC			DMR		
	SPE	CN	MNE	SPE	CN	MNE	SPE	CN	MNE
GASOLINE	90.35%	6536	0.096	87.49%	5209	0.125	89.58%	5346	0.104
SMETANA	**96.09%**	**9420**	**0.035**	**94.62%**	**9823**	**0.051**	**95.83%**	**9742**	**0.039**
NetworkBLAST-M	53.92%	7639	0.461	88.1%	5560	0.119	87.85%	5251	0.121
IsoRankN	79%	7048	0.199	83.75%	7818	0.154	85.32%	8042	0.138

**Table 2:** Performance of alignment algorithms for 4-way alignments of synthetic PPI networks (CG = crystal growth model, DMC = duplication-mutation-complementation model, DMR = duplication with random mutations model).

	CG			DMC			DMR		
	SPE	CN	MNE	SPE	CN	MNE	SPE	CN	MNE
GASOLINE	**92.75%**	10400	**0.059**	87.62%	10421	0.101	88.74%	9934	0.091
SMETANA	90.41%	**14154**	0.073	**91.06%**	**15495**	**0.07**	**93.15%**	**15255**	**0.055**
NetworkBLAST-M	31.72%	9747	0.639	44.01%	7336	0.514	56.06%	6916	0.395
IsoRankN	62.46%	5793	0.302	74.83%	8856	0.195	74.64%	9077	0.195

**Table 3:** Performance of alignment algorithms for 8-way alignments of synthetic PPI networks (CG = crystal growth model, DMC = duplication-mutation-complementation model, DMR = duplication with random mutations model).

	CG			DMC			DMR		
	SPE	CN	MNE	SPE	CN	MNE	SPE	CN	MNE
GASOLINE	**94.52%**	15359	**0.044**	**87.29%**	15735	**0.097**	88.6%	14842	0.092
SMETANA	82.93%	**17489**	0.114	83.89%	**21976**	0.102	**88.7%**	**20315**	**0.081**
NetworkBLAST-M	4.01%	5376	0.851	4.03%	5932	0.836	5.96%	6020	0.818
IsoRankN	32.09%	2433	0.485	51.74%	7112	0.305	50.84%	6677	0.305

Table 4 compares the running times of the four algorithms for each of the nine network families considered. In the pairwise case, NetworkBLAST-M and SMETANA are the fastest methods, while in the multiple case GASOLINE shows the best performances. Surprisingly, for all DMR families SMETANA performed better than GASOLINE, even in the multiple case. This is probably due to the fact that networks in DMR families are sparser than the others and GASOLINE usually works better with denser networks. This hypotheses seems to be supported by the tests performed on the real biological networks, which are two or three times denser than the synthetic ones.

**Table 4:** Running times (min) of alignment algorithms for the alignments of synthetic networks (CG = crystal growth model, DMC = duplication-mutation-complementation model, DMR = duplication with random mutations model).

	2-way			4-way			8-way		
	CG	DMC	DMR	CG	DMC	DMR	CG	DMC	DMR
GASOLINE	2.3	3.1	6.03	**4.15**	**4.15**	10.23	**11.98**	**15.83**	32.43
SMETANA	1.77	1.05	0.97	8.51	5.97	**6.18**	40.13	29.18	**29.68**
NetworkBLAST-M	**1.72**	**0.78**	**0.96**	30.85	50.62	45.35	428.93	717.36	661.22
IsoRankN		103.5	110.7	524.1	641.6	578.4	2081.4	2991.7	2350.4

Next, we investigated the effects of sequence similarities on the performances of the algorithms. Following the approach used in [23], [28], we introduced a bias term 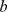 on the similarity score distribution of potential orthologs between different networks, in order to increase the differences between the similarity scores of orthologous nodes and those of non-ortologous nodes. We generated 6 different families of aligning networks, by varying 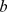 between −150 and 250. Negative values of 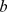 penalize sequence similarity scores, while positive values of 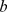 enhance them, making the alignment easier to compute. All families consist of 4 networks generated with CG model, using the phylogenetic tree of Figure 3b.

**Figure 4:**
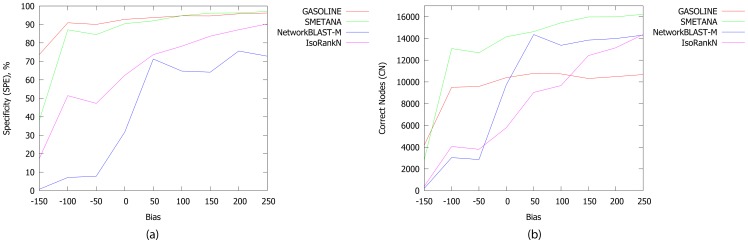
The Specificity (SPE) and Number of correct nodes (CN). SPE (a) and CN (b) for various level of bias between the similarity score distribution for orthologs and the similarity score distribution for non-orthologs.

Figure 4 reports the values of SPE and CN for different values of 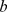. GASOLINE shows the most constant level of accuracy among all the methods, even for negative values of 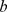. This means that our algorithm exploits topological informations well and it can produce many correct alignments even when sequence similarity scores are very noisy (73% of SPE, when 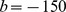). Similarly, SMETANA shows a constant level of accuracy, but its specificity is always below that of GASOLINE for non positive values of 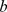. Surprisingly, for the lowest value of 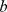 (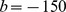), our method recovers more correct nodes than SMETANA. On the other hand, NetworkBLAST-M and IsoRankN take great advantage from the increasing bias with respect to both SPE and CN values, so they seem to strongly rely on sequence similarity scores during the computation of the alignments.

**Figure 5:**
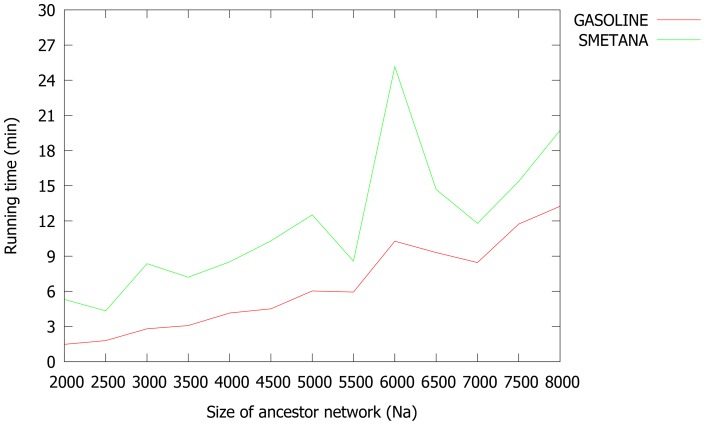
Running times of GASOLINE and SMETANA for different number of nodes (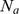) of the ancestor network.

Finally, we tested the scalability of our method, based on the size of aligning networks. We generated 7 different families, by varying the number of nodes of the ancestral network, 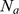 from 2000 and 5000. Again, all families consist of 4 networks generated with CG model, using the phylogenetic tree of Figure 3 (b). We performed a comparison between GASOLINE and SMETANA, which are clearly the fastest methods, as shown before. Figure 5 shows the running time for different values of 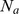. As can be seen, GASOLINE is always faster than SMETANA and generally shows less variance in running times.

### Case study 2: alignment of 6 PPI eukaryotic networks

In the second case study, we compared the four algorithms on real biological networks of 6 species (yeast, worm, fly, human, mouse and rat). Table 5 describes the features of the networks. Bit scores and BLAST E-values between all pairs of proteins belonging to different networks were computed. All pairs with E-value greater than 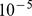 were filtered out. In order to compare the consistency and the accuracy of the algorithms, we used orthologous groups (COG, KOG and NOG), downloaded from STRING [8]. As in the previous case study, we define an equivalence class as a set of proteins of different species which are mapped together by a given algorithm. An equivalence class is claimed as correct if all the included nodes share at least one orthologous group.

**Table 5:** Features of 6 biolocial eukaryotic networks obtained from STRING.

SPECIES	# PROTEINS	# PPIs
Caenorhabditis elengans	6173	26184
Drosophila melanogaster	8624	39466
Homo sapiens	12575	86890
Mus musculus	9781	52161
Rattus norvegicus	8763	39932
Saccharomyces cerevisiae	6136	166229

To asses the performance of the algorithms, we computed specificity (SPE) and number of correct nodes (CN), and we replaced the mean normalized entropy with a different measure, the mean group consistency (MGC), defined as follows:



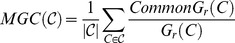



where 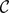 is the set of all predicted equivalence classes, 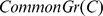 is the set of groups shared by every protein in 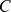 and 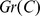 is the set of groups associated to at least one protein in 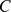.

We decided to change the consistency measure because a protein of a real biological network may be associated to more than one groups, while in the previous case study a protein was always associated to at most one group, assigned by NAPAbench during the generation of synthetic networks.

Table 6 reports the quality measures for GASOLINE, SMETANA, NetworkBlast-M and IsoRank-N in the case of pairwise and 3-way alignment. In human-mouse alignment, IsoRank-N unexpectedly failed and did not recover any conserved group. Results show that GASOLINE has much higher SPE and MGC than the compared algorithms, especially in the 3-way alignment case. Moreover, the number of correct nodes found by GASOLINE are now comparable to those of SMETANA, or even higher. Low values of CN in NetworkBlast-M are probably due to the high threshold for the minimum size of complexes (which is 5). Furthermore, NetworkBlast-M exhibit lower values for all considered metrics than GASOLINE in all tested cases.

**Table 6:** Performance of alignment methods for pairwise alignments and 3-way alignments of real biological networks (W = worm, F = fly, Y = yeast, H = human, M = mouse).

	W-Y			H-M			W-F-Y		
	SPE	CN	MGC	SPE	CN	MGC	SPE	CN	MGC
GASOLINE	**98.28%**	**4360**	**0.933**	**98.32%**	17796	**0.973**	**97.52%**	6041	**0.903**
SMETANA	82.89%	4351	0.726	96.1%	**18003**	0.939	77.79%	**6112**	0.662
NetworkBLAST-M	93.77%	2545	0.742	81.2%	6747	0.713	84.13%	3178	0.595
IsoRankN	67.88%	3900	0.601	0%	0	0	56.29%	4526	0.485

Such results are confirmed for the alignment of 4, 5 and 6 species (Table 7). It is worth noting that the specificity of GASOLINE remains very high (around 95%) and the differences between GASOLINE and the other methods increase (around 20% specificity more than the second best algorithm, SMETANA). In this case, quality measures are not reported for IsoRank-N because of its high running time (more than 2 days of computation).

**Table 7:** Performance of alignment methods for 4-way, 5-way and 6-way alignments of real biological networks (W = worm, F = fly, Y = yeast, H = human, M = mouse, R = rat).

	W-H-M-Y			W-F-H-M-Y			W-F-H-M-R-Y		
	SPE	CN	MGC	SPE	CN	MGC	SPE	CN	MGC
GASOLINE	**95.5%**	**7954**	**0.861**	**94.82%**	9166	**0.847**	**93.8%**	10385	**0.822**
SMETANA	76.95%	7913	0.653	75.19%	**9368**	0.631	73.7%	**10677**	0.612
NetworkBLAST-M	63%	4651	0.303	60.95%	5343	0.268	51.62%	5829	0.228

To sum up, the performance results of GASOLINE in the context of real biological networks are superior to those of synthetic networks, with respect to both specificity and number of correct nodes, which is related to the sensitivity of the algorithm. Moreover, the values of CN are very close to or even higher than SMETANA, though the latter is a global alignment method.

A further comparison between GASOLINE and NetworkBlast-M was made to assess the statistical and biological significance of complexes found by both methods in the alignment of 6 species. We annotated aligned proteins with GO terms (cellular components, processes and functions), taken from BioDBNet [35]. We computed, for every GO category in each complex of the alignments, a 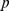-value based on the hypergeometric distribution. Finally, 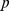-values have been corrected by applying FDR correction for multiple hypotheses testing, with 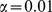.

Table 8 shows the 10 best complexes identified by GASOLINE, sorted by their size and 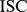 score. The number of enriched GO categories together with the ranking of the corresponding complexes found by NetworkBlast-M are reported. The table shows that the best results found by GASOLINE are also among the best results identified by NetworkBlast-M.

**Table 8:** Best 10 complexes found by GASOLINE.

RANK	DESCR	SIZE	ISC	GOs	NetBlast RANK
1	Large and small subunit	59	85.6%	16	10, 12
	of ribosomes in the cytosol				14, 15
2	Spliceosome	40	87.1%	13	5, 9
3	Proteasome	32	95%	17	2, 3
4	Ribosome biogenesis	25	89.2%	11	4, 16
	in the nucleolus				
5	Protein serine/threonine	25	75.6%	19	34, 35
	kinase activity				
6	DNA repair complex	24	92.5%	39	18
7	SSU processome	22	96.4%	4	1
8	DNA directed	21	94.2%	13	6, 7
	RNA polymerase				
9	Vesicle-mediated transport	20	85.5%	20	19
10	Prefoldin complex	19	90.6%	2	37

GASOLINE found more complexes than NetworkBlast-M (46 vs 45). However, most of the results are common to both methods. Nine small complexes (5–7 proteins) have been identified only by GASOLINE and eight small complexes (5–10 proteins) have been recovered only by NetworkBlast-M.

Some of the complexes are correctly split by GASOLINE and wrongly joined in NetworkBlast-M, while other complexes in GASOLINE are actually smaller than the corresponding ones in NetworkBlast-M. This is probably due to the different scoring functions used by the two methods.

All complexes returned by NetworkBlast-M can include non 1-to-1 mapping between proteins of different networks. However, these have a fixed maximum size of 15 proteins. This is a serious limitation in the context of local alignment of biological networks since real biological complexes can be actually bigger [36]. Table 9 shows that the most significant GO categories found by GASOLINE and NetworkBlast-M for the Proteasome complex have similar significant 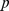-values. Nevertheless, the Proteasome complex found by GASOLINE includes more proteins than the one found by NetworkBlast-M (32 vs 15 proteins).

**Table 9:** GO enriched categories related to the Proteasome complex.

GO category	GASOLINE	NetworkBlast-M
GO:0000502	5.551E-17	3.775E-16
GO:0005839	3.701E-17	1.110E-16
GO:0019773	1.199E-15	8.882E-17
GO:0051603	1.480E-16	2.405E-16
GO:0004298	5.551E-17	9.252E-17

In Table 10 we report the running times of GASOLINE, SMETANA, NetworkBlast-M and IsoRank-N. In the case of pairwise and 3-way alignment, NetworkBlast-M is faster than GASOLINE. However, GASOLINE clearly outperforms NetworkBlast-M and the other algorithms in the multiple case scaling well with the number of networks.

**Table 10:** Running times of GASOLINE, SMETANA, NetworkBlast-M and IsoRank-N.

Alignment	GASOLINE	SMETANA	NetworkBlast-M	IsoRank-N
W-Y	154 sec	125 sec	**59 sec**	54460 sec
H-M	890 sec	1587 sec	**205 sec**	16620 sec
W-F-Y	**175** sec	351 sec	281 sec	148320 sec
W-H-M-Y	**409 sec**	6310 sec	4854 sec	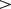 2 days
W-F-H-M-Y	**533 sec**	13380 sec	5999 sec	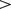 2 days
All networks	**666 sec**	22185 sec	12487 sec	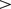 2 days

### Case study 3: alignment of 25 vertebrata PPI networks

In the last case study, we collected a dataset of 25 vertebrata biological networks. Table 11 describes the features of these networks. We ran GASOLINE with higher values of 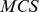 and 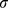 (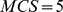, 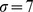), for computational reasons due to the high number of aligned networks. We found 36 complexes conserved in all species. Table 12 lists the 10 highest-scored ones, together with the number of significantly enriched GO categories.

**Table 11:** Features of 25 biological eukaryotic networks downloaded from STRING.

SPECIES	# PROTEINS	# PPIs
Anolis carolinensis	6510	31135
Bos taurus	8474	42234
Canis familiaris	8440	42239
Cavia porcellus	8185	42208
Danio rerio	5720	25732
Dasypus novemcinctus	6850	30495
Equus caballus	8144	40703
Felis catus	7200	32547
Gallus gallus	6409	29534
Gasterosteus aculeatus	6018	28276
Homo sapiens	12575	86890
Macaca mulatta	8787	41460
Monodelphis domestica	7800	38002
Mus musculus	9781	52161
Ornithorhynchus anatinus	6035	26467
Oryctolagus cuniculus	8010	39304
Oryzias latipes	5754	26880
Pan troglodytes	8677	44263
Pongo pygmaeus	8551	43984
Rattus norvegicus	8763	39932
Sus scrofa	6752	29852
Taeniopygia guttata	6271	28791
Takifugu rubipres	5872	27077
Tetraodon nigroviridis	5779	25730
Xenopus tropicalis	6153	29769

**Table 12:** Best 10 conserved complexes found by GASOLINE for the alignment of 25 vertebrata PPI networks.

RANK	DESCR	SIZE	ISC	GOs
1	Protein serine/threonine kinase activity complex	26	86.1%	19
2	Proteasome	20	91.3%	14
3	Nuclear receptor DNA complex	16	78.7%	13
4	Histone deacetylase complex	14	85.4%	14
5	Vesicle-mediated transport	13	86.5%	10
6	Cyclin-dependent kinase complex	13	85.9%	8
7	Chaperonin-containing T-complex	13	85.5%	8
8	DNA directed RNA polymerase II	12	94.3%	8
9	Eukaryotic translation initiation factor 3	12	91.8%	5
10	Spliceosome	11	92.6%	5

Most of the complexes found by GASOLINE in the second case study are also present in this third one. However they are smaller here (i.e. spliceosome), due to (i) the higher number of aligned networks; (ii) incompleteness of PPI networks data in some species. GASOLINE took 2250 seconds (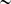
38 minutes) to perform the alignment of all 25 vertebrata PPI networks.

**Figure 6:**
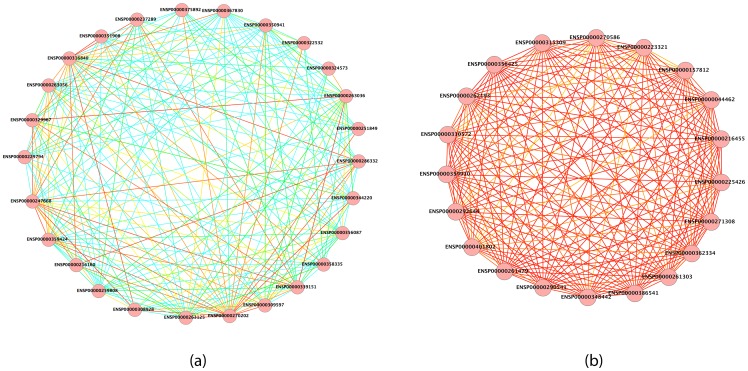
Meta-graph of complexes found by GASOLINE for the alignment of 25 PPI vertebrata networks. (a) Chaperonin complex, (b) Proteasome complex. Cyan indicates low conservation, green medium, yellow high and red very high.

We also analyzed phylogenetic relations among corresponding proteins of distant species in local alignments. Largest and most conserved complexes returned by GASOLINE, the proteasome and the chaperonin, were considered. We represented the conserved cluster of interactions as a single meta-graph (Figure 6), where nodes are classes of aligned proteins (one for each species) and edges are colored according to the conservation extent of the corresponding interaction.

In Figure 6 (a) we depict the meta-graph of Chaperonin complex, whereas in Figure 6 (b) we present the Proteasome complex. In both complexes, we can observe the presence of a big core of highly conserved protein interactions. This may represent a sort of ancestral complex from which all the species-specific complexes have differently evolved.

## Conclusions

GASOLINE is a new multiple local network alignment algorithm based on Gibbs Sampling Experimental analysis clearly shows that GASOLINE outperforms state-of-art systems such as: SMETANA, NetworkBlast-M and IsoRank-N on real biological networks. GASOLINE does not allow non 1-to-1 mapping, although this can be viewed as a limitation, the results clearly show that such a restriction is capable to produce more reliable results than methods implementing many-to-many mapping. Furthermore, GASOLINE is able to find new complexes and unlikely NetworkBlast-M it correctly splits intersecting complexes and it does not have any size limitation. Finally it is clearly faster than all the compare systems. GASOLINE is a very general method which can be applied to all kinds of large networks by a suitable choice of label and topology similarities. Applications in the field of protein structure comparison and social networks are under development.

## Supporting Information

File S1 **Contains supporting material on GASOLINE asymptotic complexity.**
(PDF)
