# Supplementary material for: GASOLINE: a Greedy And Stochastic algorithm for Optimal Local multiple alignment of Interaction NEtworks
Source: PLoS One. 2014 Jun 9;9(6):e98750. doi: 10.1371/journal.pone.0098750 (PMC4049608; doi:10.1371/journal.pone.0098750)
Supplement: File S1 — Contains supporting material on GASOLINE asymptotic complexity. (PDF) [file pone.0098750.s001.pdf]

Supplementary Material for "GASOLINE: a Greedy And Stochastic algorithm for Optimal Local multiple alignment of Interaction NEtworks"

Giovanni Micale[1,§], Alfredo Pulvirenti[2,§,*], Rosalba Giugno[2], Alfredo Ferro[2]
1Department of Computer Science, University of Pisa, Pisa, Italy.
2Department of Clinical and Molecular Biomedicine, University of Catania, Catania, Italy.
***E-mail: Corresponding apulvirenti@dmi.unict.it**
§**Equal contributors**

Computational complexity of GASOLINE

In this section we analyze the time complexity of GASOLINE. To simplify the analysis, we suppose that the size of the complexes returned at the end of each execution of GASOLINE is W, we suppose also that we have N input networks with the same number of nodes, n, and edges m. We define also the following variables:

- γ_s: the number of Gibbs sampling iterations of in the bootstrap phase;
- γ_e: the number of Gibbs sampling iterations in each extension step of the *iterative phase*;
- k: the average degree of a node ($k = \frac{m}{n}$);
- γ_i: the number of iterations of the iterative phase;
- γ_x: the number of executions of GASOLINE.

Time complexity will be expressed as a function of n, N and W. Through the analysis, we will assume that the generation of random numbers and the computation of the orthology score between two proteins are done in constant time $O(1)$.

First, let's analyze the bootstrap initial phase, whose goal is to find an optimal alignment of protein seeds. The generation of the initial alignment requires time $O(N)$. The computation of transition probabilities for all the proteins of the selected network at each iteration of Gibbs sampling costs $O(nN)$, while the computation of the alignment score requires $O(N)$.

Therefore, the time complexity of the initial phase is:

$$T_{boot}(n) = O(N) + \gamma_s \times O(nN) = O(\gamma_s nN)$$

Since, in practice, $N << n$ we can write:

$$T_{boot}(n) = O(\gamma_s n) \quad (1)$$

Now, let's consider the iterative phase, which removes and adds nodes to the current local alignment iteratively.

Suppose, for simplicity, that the alignment grows in the following way: at the beginning the size of aligned complexes increases from 1 up to W, then in the following extension and removal steps it switches from W to W-1 and viceversa.

The last assumption fits quite well the behavior of GASOLINE in the context of real biological networks, since our algorithm yields an alignment of complexes of a certain size and then tries to adjust it by replacing bad parts according to a goodness score.

Let's start with the extension step, which can be divided into three phases:

1. The computation of seeds' adjacent nodes;

2. The execution of Gibbs sampling;

3. The extension of seeds.

Let's suppose that networks are represented through adjacency lists. Under this assumption, the adjacent of a node can be found in $O(k)$ time. As regards Gibbs sampling, the generation of the initial alignment requires $O(N)$ time.

The computation of the transition probabilities and the alignment score depends on the size of seeds. Let L the current size of the aligned complexes. The transition probability of a protein is computed as the product of two components: orthology similarity score and topology similarity score.

Computing the orthology score for a protein of the selected network at each iteration of Gibbs sampling costs $O(N)$ as in the bootstrap phase.

In order to compute topology scores efficiently, topology vectors are built before starting Gibbs sampling, for all seed's adjacent nodes of all aligning networks. The construction of topology vector of a single protein can be done in $O(L)$ time, assuming that adjacent lists are implemented by using hash tables with buckets, thus providing constant-time access (in average) to an element of the list. So, the overall cost of building topology vectors is $N \times O(kL^2)$, supposing that the total number of a seed's adjacent nodes is $O(kL)$. Under this assumptions, the orthology score of a protein can be computed in $O(NL)$ time and the transition probability for all the proteins of the selected network requires $O(nNL)$ time. Finally, the computation of the alignment score requires $O(NL)$ time.

Summing up, the overall cost of the extension step we obtain:

$$T_{ext}(n) = N \times O(kL^2) + O(N) + \gamma_e \times O(nNL)$$

Assume $N << n$ and $k << n$ we can rewrite the equation as:

$$T_{ext}(n) = O(L^2 + \gamma_e nL) \tag{2}$$

Since in the worst case $L = O(n)$ we can deduce that:

$$T_{ext}(n) = O(\gamma_x nL) \tag{3}$$

The removal step simply consists in computing the minimum value of a function (*Goodness* score) overall the L sets of aligned proteins in the current alignment. For each set, the *Goodness* score can be evaluated in $O(NL)$, which is the time required to compute the internal degree of all the proteins within the set. So, the cost of removal step is:

$$T_{rem}(n) = O(NL^2) = O(L^2) \tag{4}$$

Assuming that the extension step is performed $W-1$ times at the beginning and $\gamma_i - 1$ times later on and the removal step is executed γ_i times, the overall cost of the iterative phase is:

$$\begin{aligned} T_{iter}(n) = & \sum_{i=1}^{W-1} O(\gamma_e nL) + \\ & + (\gamma_i - 1) \times O(\gamma_e n(W-1)) + \\ & + (\gamma_i - 1) \times O((W-1)^2) \end{aligned}$$

We can assume, without loss of generality, that $W = O(\gamma_i)$ and $W << n$, so:

$$T_{iter}(n) = O(\gamma_i \gamma_e nW) \tag{5}$$

Finally, all preprocessing steps can be performed in linear time, by considering the degree and the number of orthologous proteins for the proteins of all networks. Post-processing phase consists in filtering highly overlapping complexes and can be done in constant time.

By combining equations (1) and (4) and considering preprocessing operations, the overall cost of γ_e executions of GASOLINE is:

$$\begin{aligned} T(n) &= O(n) + \gamma_x \times [T_{boot}(n) + T_{iter}(n)] \\ &= O(n) + \gamma_x \times [O(\gamma_s n) + O(\gamma_i \gamma_e nW)] \end{aligned}$$

From the results of the analysis, it follows that the running time of GASOLINE is polynomial in n. In fact, γ_x is at most equal to n since at each execution of the algorithm different protein seeds are considered. Moreover, in all applications, γ_s and γ_e are in the range 200-400 and can be considered constant. Therefore, the final complexity is $O(n^2 W)$. We can distinguish three cases:

1. If networks are very similar, then the average size W of complexes found in each execution is high, so $W = O(n)$ and the algorithm requires $O(n^3)$ time. This is the worst case;

2. If networks are very distantly related, then $W = O(\sqrt{n})$ and the running time is $O(n^{2.5})$ this is the average case.

3. If W is independent to the size of networks we can suppose its size constant $W = O(1)$. Therefore, the running time will be $O(n^2)$ that is, the best case of our algorithm.
